# Effect of national holidays on health outcomes of patients receiving peritoneal dialysis in a single center over a ten-year period

**DOI:** 10.1080/0886022X.2022.2153697

**Published:** 2023-01-16

**Authors:** Wei Yang, Li Zeng, Huibin Yang, Fujia Guo, Dan Zhou, Wenting Cui, Shuran Wu, Cong Chen, Jiayao Zhao, Weidong Wang, Ning Yang, Hongli Lin, Longkai Li

**Affiliations:** a Department of Nephrology, Liaoning Translational Medicine Center of Nephrology, First Affiliated Hospital of Dalian Medical University, Dalian, China; b First Affiliated Hospital, Dalian Medical University, Dalian, China; c College of Integrative Medicine, Dalian Medical University, Dalian, China; d Graduate School, Dalian Medical University, Dalian, China

**Keywords:** Peritoneal dialysis, holiday, hospitalization, death, peritonitis

## Abstract

**Background:**

National holidays are associated with high mortality in some diseases, but little is known about patients undergoing peritoneal dialysis (PD). The research aimed to investigate the impact of national holidays on the health outcomes of PD patients.

**Methods:**

Over ten years, all episodes of unplanned hospitalization, death, and peritonitis in PD patients were collected in our center. Seven national holidays in China were chosen, and non-holiday days were selected as the control period. The effect of national holidays was observed by comparing the hospitalization, death, and peritonitis rates between holiday and non-holiday groups.

**Results:**

There were 297 events in all holiday periods and 1247 in non-holiday periods. There is no significant difference in hospitalization rate between holiday and non-holiday groups (32.4% ± 6.4% vs. 29.2% ± 3.4%, *p* = 0.175). So is the death rate [6.3% (4.8–12.3%) vs.5.0% (4.2–8.9%), *p* = 0.324] and peritonitis rate [0.19 (0.13–0.53) vs. 0.22 (0.18–0.27), *p* = 0.445] between the two groups. Significant differences were observed in the distribution of peritonitis causes between the two groups (*p =* 0.017). The rate of secondary to other infections in the holiday group was significantly higher than in the non-holiday group (25.0 vs. 10.3%, *p* = 0.015).

**Conclusion:**

Our study suggested no national holiday effect on health outcomes of PD patients based on ten-year data in our center.

## Introduction

‘National holiday effect’ has been proven to significantly affect the prognosis of patients with many diseases (acute myocardial infarction and stroke) and increase mortality [1,2]. A Canadian study recorded 188 excess rehospitalizations and 26 excess deaths per 100,000 patients discharged during the Christmas holiday [3]. The risk of myocardial infarction was increased by 15% and 37% during Christmas or New Year holidays and Christmas Eve in Sweden [4]. A study on stroke patients also demonstrated that in-hospital mortality, 7-day mortality, and 30-day mortality during the Chinese New Year holiday increased by 20%, 33% and 21%, respectively [5]. The studies mentioned above mainly focused on acute diseases; however, there were few studies about the holiday effect on chronic diseases, and little is known about patients receiving peritoneal dialysis (PD). Therefore, it is necessary to explore the effect of national holidays on the prognosis of PD patients to deepen our understanding of the holiday effect.

Although few studies focused on the ‘holiday effect’ on chronic diseases, the results were inconsistent. A study on patients with type 1 diabetes showed that their adherence to metformin was reduced during South Australian official public holidays, resulting in a higher hospitalization rate and mortality rate [6]. However, a study in Taiwan suggested that there was no significant increase in the 30-day mortality rate among those hospitalized during the Chinese New Year holiday in patients with chronic diseases such as chronic obstructive pulmonary disease [7]. Peritoneal dialysis patients, a large population with different characteristics from acute diseases, have long chronic duration [8,9] and should control their conditions all the time, which is crucial for PD patients with fewer follow-ups compared to hemodialysis patients. However, it is still being determined whether there is a national holiday effect on PD patients. Therefore, there is a great need for a full exploration of the national holiday effect on the health outcomes of PD patients.

PD patients need regular follow-ups and guidance to prevent and control medical negligence and complications [10,11]. Decreased follow-up may lead to untimely and less early help and instruction from medical staff for PD patients [3], and there may be increased episodes of hospitalization, deaths, and peritonitis in PD patients during the national holiday period. To date, there is no study on the national holiday effect on PD patients. In the present study, we performed a single-center retrospective study to evaluate the potential impact of national holidays on the health outcomes of PD patients.

## Methods

### Study design

It is a retrospective study on PD patients in the Department of Nephrology, First Affiliated Hospital of Dalian Medical University, China. All PD patients with unplanned hospitalization, death, and peritonitis were enrolled from 1 January 2009, to 31 December 2019. The study was carried out following the Declaration of Helsinki. Related data about the personal identity of patients were treated anonymously.

### Study periods

According to the General Office of the State Council of the People’s Republic of China, there are seven official holidays in China, including New Year, Chinese New Year, Tomb-Sweeping Day, Labor Day, Dragon Boat Festival, Mid-Autumn Festival, and National Day. Their holiday periods are seven days for Chinese New Year, National Day, three days for New Year, Tomb-Sweeping Day, Labor Day, Dragon Boat Festival, and Mid-Autumn Festival, respectively [12]. Considering that part of the effects of holidays on diseases may be reflected after the holidays [2], we extended the holiday period by four or seven days. Holiday periods were 63 days in one year as follows, New Year (7 days), Chinese New Year (14 days), Labor Day (7 days), Tomb-Sweeping Day (7 days), Dragon Boat Festival (7 days), Mid-Autumn Festival (7 days) and National Day (14 days). The left 302 days were selected as the non-holiday period. The date of holidays and the Chinese lunar calendar corresponding to each festival were presented in Supplemental Table 1.

### Data collection and clinical outcomes

Most of the clinical data were obtained from the electronic medical record of our hospital, including gender, age, PD vintage, etiology of end-stage of renal disease (ESRD), hospitalization data, death, and peritonitis. In addition, there are complete follow-up records of PD patients about hospitalization, death, peritonitis, total weekly Kt/V(urea), and creatinine clearance (Ccr) in our PD center. All the causes of peritonitis were also collected. The primary outcomes were the rates of hospitalizations, deaths, and peritonitis in holiday and non-holiday periods.

### Diagnosis of peritonitis

According to the guidelines of peritonitis of International Society for Peritoneal Dialysis (ISPD) in 2022 [13], peritonitis was defined as when at least two of the following conditions exist: (1) consistent with the clinical features of peritonitis, that is, abdominal pain or cloudy dialysis effluent; (2) The white blood cell count of the dialysis effluent is >100/μL or >0.1 × 10^9^/L (after a dwell time of at least two hours), and polymorphonuclear leukocytes is >50%; (3) positive dialysis effluent culture.

### Statistical analysis

The data were presented using mean ± standard deviation for normally distributed data and numbers (*n*) and percentages (%) for categorical variables. Baseline demographic differences between holiday and non-holiday periods were analyzed using *t*-tests for normally distributed continuous data and the chi-squared (*χ*^2^) tests for categorical data. *T*-tests and rank sum tests were used to compare the incidence of hospitalization, death, and peritonitis between the holiday and non-holiday groups. The incidence rate is defined as rate = number of episodes/days (year) [14–16]. The Chi-square test was used to compare the causes of peritonitis between holidays and non-holidays. Statistical significance was defined as *p*-value <0.05. All data were analyzed using SPSS 22.0 (Statistical Package for the Social Sciences software, IBM Corp., Armonk, NY, USA).

## Results

### Patient characteristics

All the events (hospitalization, death, and peritonitis) in holiday and non-holiday periods were identified in our department between 1 January 2009, and 31 December 2019, including 297 holiday episodes and 1247 non-holiday episodes. The characteristics of each group are summarized in Table 1. In terms of the ESRD etiology, diabetic nephropathy (683, 44.67%) ranked first, followed by glomerulonephritis (527, 34.47%) and other causes of ESRD (319, 20.86%). There were no significant differences between the holiday and non-holiday groups in age, gender, PD vintage, etiology of ESRD, Kt/v, and Ccr. (Table 1).

**Table 1.** Baseline demographics of study population.

Characteristic	Holiday group	Non-holiday group	*p* Value
Patients (*n*)	247	1034	
Female (*n*, %)	99 (40.08%)	412 (39.85%)	0.946
Age (yrs)	59.87±15.63	60.67±14.48	0.441
Dialysis vintage (ms)	25.14±23.73	25.73±24.43	0.734
Etiology of ESRD			0.543
Diabetic nephropathy	105 (42.51%)	465 (44.87%)	
Glomerulonephritis	85 (35.63%)	356 (34.24%)	
Others	57 (21.86%)	213 (20.89%)	
Kt/v	1.67±0.55	1.68±0.56	0.851
Ccr	62.21±46.60	59.88±31.05	0.448

The data were expressed as the median (range) and mean ± standard deviation.

ESRD: end-stage renal disease; Ccr: creatinine clearance.

### Effect of holiday on hospitalization, death, and peritonitis

In order to observe whether there was a holiday effect on the health outcomes of PD patients, we first compared the difference in the event rates between holiday and non-holiday groups (Table 2). There is no significant difference in hospitalization rate between holiday and non-holiday groups, although it was slightly higher in the holiday group (32.4% ± 6.4% vs. 29.2% ± 3.4%, *p* = 0.175). The death rate was 6.3% (4.8–12.3%) in the holiday group and 5.0% (4.2–8.9%) in the non-holiday group, but no significant difference was observed between the two groups (*p* = 0.324). The peritonitis rate was also analyzed, it was 0.19 (0.13–0.53) episodes per patient-year in the holiday group and 0.22 (0.18–0.27) episodes per patient-year in the non-holiday group; however, there was no significant difference between the two groups (*p* = 0.445).

**Table 2.** Comparison of hospitalization, mortality and peritonitis between holiday and non-holiday groups.

Group and events	No. of events	Event rate(%)	*p* Value*
Holiday group			
Hospitalization	204	32.4% ± 6.4%	0.175
Death	53	6.3% (4.8–12.3%)	0.324
Peritonitis	40	0.19 (0.13–0.53)^#^	0.445
Non-holiday group			
Hospitalization	881	29.2% ± 3.4%	–
Death	186	5.0% (4.2–8.9%)	–
Peritonitis	180	0.22 (0.18–0.27)^#^	–

The data were expressed as the median (range) and mean ± standard deviation.

*p* Value*, the comparison of hospitalization, death and peritonitis between holiday and non-holiday groups.

^#^Patient-year of peritonitis.

### Comparison of peritonitis causes between two groups

In the analysis of the causes of peritonitis, enteric causes, secondary to other infections, touch contamination, and others were included. Although enteric causes ranked first in both groups, there were more episodes due to secondary to other infections in the holiday group than those in the non-holiday group (10, 25% vs. 19, 10.56%, *p =* 0.015). There were significant differences in the distribution causes between holiday and non-holiday groups (*p =* 0.017) (Supplementary Table 2).

## Discussion

In the present study, all the ten-year data in our center were collected to evaluate the ‘holiday effect’ on health outcomes of PD patients, including hospitalization, death, and peritonitis. Unlike most studies, there was no obvious evidence of the ‘holiday effect’ on hospitalization, death, and peritonitis during Chinese national holidays.

Previous studies mainly focused on the impact of the ‘holiday effect’ on acute diseases, such as acute myocardial infarction (AMI) [17] and pulmonary embolism (PE)[18]. The AMI mortality rate was higher on Christmas and New Year [2] and Chinese National Day[17]. The Taiwan study demonstrated that patients admitted to hospitals for PE were associated with increased mortality during the Chinese New Year holiday [18]. However, few studies focused on the ‘holiday effect’ on chronic diseases. In the present study, we aimed at the ‘holiday effect’ on health outcomes of PD patients, and the results showed no ‘holiday effect’ on these patients in hospitalization, death, and peritonitis during Chinese official holidays. The reason for the different effects of the holiday may be different types of diseases (acute and chronic diseases). PD patients were well trained at the beginning of renal replacement treatment to control their diet, evaluate their condition frequently, and communicate with the medical staff if needed, especially on weekends and holidays. According to ISPD guidelines [13], a syllabus for teaching peritoneal dialysis to patients and caregivers was the essential section. In addition, frequent and scheduled retraining for PD patients and caregivers was also crucial for patients’ prognosis [19,20]. Therefore, PD patients have to focus on their health all the time because of their unique disease.

Interestingly, there were not dramatically decreased episodes of peritonitis during holidays in the present study, which is inconsistent with one previous study on the weekend effect. A study from Australia and New Zealand found that the number of patients with peritonitis on weekends decreased. The reason may be that patients had more time to perform PD on weekends carefully and reduced the possibility of making mistakes [21]. However, there were significant differences in the distribution causes between holiday and non-holiday groups. The reason may be that the lifestyle is different on holiday from that on weekends. PD patients may have more chances to visit relatives or friends during holidays. During this process, there may be more festival activities, insufficient sleep, and increased emotional stress. Lifestyle changes can easily result in the deterioration of their fragile systemic condition [22], resulting in a systemic infection that leads to peritonitis. This reason may be why there were more peritonitis episodes secondary to other infections during the holiday than in non-holiday periods.

There are some strengths and limitations to this study. It is the first study to evaluate the ‘holiday effect’ on the health outcomes of PD patients. The study covered all the Chinese holidays, and the holiday period was extended enough to reflect the holiday effect, making our research more comprehensive. However, as a single-center study, although we have collected data for ten years, the study’s results still cannot represent the ‘holiday effect’ in all regions. As a retrospective study, we could not obtain detailed clinical information about our patients, which may affect the research results. Therefore, multiple-center and large-sample research on the ‘holiday effect’ is in great need in the future.

In conclusion, our study showed no ‘holiday effect’ on hospitalization, death, and peritonitis in PD patients in our center. Further studies are needed in multiple centers for extended observation periods to explore the ‘holiday effect’ in PD patients.

## Supplementary Material

Supplemental Material

## References

[1] Kloner RA, Poole WK, Perritt RL. When throughout the year is coronary death most likely to occur? A 12-year population-based analysis of more than 220 000 cases. Circulation. 1999;100(15):1630–1634. 10517734 10.1161/01.cir.100.15.1630

[2] Phillips DP, Jarvinen JR, Abramson IS, et al. Cardiac mortality is higher around Christmas and new year’s than at any other time: the holidays as a risk factor for death. Circulation. 2004;110(25):3781–3788. 15596560 10.1161/01.CIR.0000151424.02045.F7

[3] Lapointe-Shaw L, Austin PC, Ivers NM, et al. Death and readmissions after hospital discharge during the December holiday period: cohort study. BMJ. 2018;363:k4481. 30530782 10.1136/bmj.k4481 PMC6287120

[4] Mohammad MA, Karlsson S, Haddad J, et al. Christmas, national holidays, sport events, and time factors as triggers of acute myocardial infarction: SWEDEHEART observational study 1998-2013. BMJ. 2018;363:k4811. 30541902 10.1136/bmj.k4811 PMC6289164

[5] Huang HK, Chang WC, Hsu JY, et al. Holiday season and weekend effects on stroke mortality: a nationwide cohort study controlling for stroke severity. J Am Heart Assoc. 2019;8(8):e011888. 30973048 10.1161/JAHA.118.011888 PMC6507216

[6] Leggett C, Giles L, Anderson JJA, et al. Adherence to metformin is reduced during school holidays and weekends in children with type 1 diabetes participating in a randomised controlled trial. Arch Dis Child. 2019;104(9):890–894. 31079072 10.1136/archdischild-2018-316303

[7] Lin SM, Wang JH, Huang LK, et al. Does the 'Chinese new year effect’ exist? Hospital mortality in patients admitted to internal medicine departments during official consecutive holidays: a nationwide population-based cohort study. BMJ Open. 2019;9(4):e025762. 10.1136/bmjopen-2018-025762 PMC6500326 31005924

[8] Wouk N. End-stage renal disease: medical management. Am Fam Physician. 2021;104(5):493–499. 34783494

[9] Kee YK, Park JT, Yoon CY, et al. Characteristics and clinical outcomes of end-stage renal disease patients on peritoneal dialysis for over 15 years: a single-center experience. Perit Dial Int. 2017;37(5):535–541. 28546366 10.3747/pdi.2016.00227

[10] Schaepe C, Bergjan M. Educational interventions in peritoneal dialysis: a narrative review of the literature. Int J Nurs Stud. 2015;52(4):882–898. 25616708 10.1016/j.ijnurstu.2014.12.009

[11] Van Biesen W, Verger C, Heaf J, IPOD-PD Study Group, et al. Evolution over time of volume status and PD-related practice patterns in an incident peritoneal dialysis cohort. Clin J Am Soc Nephrol. 2019;14(6):882–893. 31123180 10.2215/CJN.11590918 PMC6556715

[12] Tang L, Chen PF, Hu XQ, et al. Effect of Chinese national holidays and weekends versus weekday admission on clinical outcomes in patients with STEMI undergoing primary PCI. J Geriatr Cardiol. 2017;14(10):604–613. 29238361 10.11909/j.issn.1671-5411.2017.10.003 PMC5721195

[13] Li PK, Chow KM, Cho Y, et al. ISPD peritonitis guideline recommendations: 2022 update on prevention and treatment. Perit Dial Int. 2022;42(2):110–153. 35264029 10.1177/08968608221080586

[14] Rodriguez-Jato Q, Pereira AR, Batalla A, et al. Hospitalization in patients with psoriasis: impact of biological therapies on temporal evolution. J Drugs Dermatol. 2021;20(2):208–214. 33538560 10.36849/JDD.4931

[15] Chen W, Zheng R, Zhang S, et al. Cancer incidence and mortality in China, 2013. Cancer Lett. 2017;401:63–71. 28476483 10.1016/j.canlet.2017.04.024

[16] Sakurada T, Fujishima R, Yamada S, et al. Seasonality of peritoneal dialysis-related peritonitis in Japan: a single-center, 10-year study. Clin Exp Nephrol. 2021;25(1):52–57. 32783172 10.1007/s10157-020-01953-1

[17] Lin X, Green JC, Xian H, et al. Holiday and weekend effects on mortality for acute myocardial infarction in Shanxi, China: a cross-sectional study. Int J Public Health. 2020;65(6):847–857. 32737560 10.1007/s00038-020-01443-x

[18] Hung DP, Lin SM, Liu PP, et al. Evaluating the "holiday season effect" of hospital care on the risk of mortality from pulmonary embolism: a nationwide analysis in Taiwan. Sci Rep. 2021;11(1):19376. 34588561 10.1038/s41598-021-98845-5 PMC8481409

[19] Figueiredo AE, Bernardini J, Bowes E, et al. A syllabus for teaching peritoneal dialysis to patients and caregivers. Perit Dial Int. 2016;36(6):592–605. 26917664 10.3747/pdi.2015.00277 PMC5174866

[20] Zhang L, Hawley CM, Johnson DW. Focus on peritoneal dialysis training: working to decrease peritonitis rates. Nephrol Dial Transplant. 2016;31(2):214–222. 26908816 10.1093/ndt/gfu403

[21] Johnson DW, Clayton P, Cho Y, et al. Weekend compared with weekday presentations of peritoneal dialysis-associated peritonitis. Perit Dial Int. 2012;32(5):516–524. 22302768 10.3747/pdi.2011.00169 PMC3524873

[22] Jahromi MG, Goudarzi R, Yazdi-Feyzabadi V, et al. Effect of new year holidays on hospital mortality: a time series study. Int J Emerg Med. 2019;12(1):20. 31455232 10.1186/s12245-019-0243-x PMC6712866

