# Supplementary material for: Effect of national holidays on health outcomes of patients receiving peritoneal dialysis in a single center over a ten-year period
Source: Ren Fail. 2023 Jan 16;45(1):2153697. doi: 10.1080/0886022X.2022.2153697 (PMC9848312; doi:10.1080/0886022X.2022.2153697)
Supplement: Supplemental Material [file IRNF_A_2153697_SM9540.pdf]

Supplemental Table 1 The specific time or month corresponding to each holiday.

Holiday	Chinese lunar calendar	Gregorian calendar
New Year	—	January 1st
Chinese New Year	January 1st	January or February
Tomb-Sweeping Day	—	April 4th to April 6th
Labor Day	—	May 1st
Dragon Boat Festival	May 5th	June
Mid-Autumn Festival	August 15th	September or October
National Day	—	October 1st

Information Classification: General

Supplementary Table 2 Comparison of causes of peritonitis in holiday and non-holiday groups.

Peritonitis causes	Holiday group	Non-holiday group	*p* Value
Enteric causes	20(50.00%)	76(42.53%)	0.370
Secondary to other infection	10(25.00%)	19(10.34%)	***0.015***
Touch contamination	2(5.00%)	32(17.24%)	0.075
Other causes	8(20.00%)	53(29.89%)	0.227
Number of all episodes	40(100%)	180(100%)	***0.017***

Bold and italic indicate $p < 0.05$.

Information Classification: General
